# The role of *Bifidobacterium* in longevity and the future of probiotics

**DOI:** 10.1007/s10068-024-01631-y

**Published:** 2024-07-11

**Authors:** Seockmo Ku, Md Ariful Haque, Min Ji Jang, Jaehyun Ahn, Deokyeong Choe, Jong Ik Jeon, Myeong Soo Park

**Affiliations:** 1 Department of Food Science and Technology, Texas A&M University, College Station, TX 77843 USA; 2 Department of Agricultural Leadership, Education and Communications, Texas A&M University, College Station, TX 77843 USA; 3 School of Food Science and Biotechnology, Kyungpook National University, Daegu, 41566 Korea; 4 Research Center, BIFIDO Co., Ltd, Hongcheon, 25117 South Korea

**Keywords:** Probiotics, Bifidobacterium, Longevity, Microbiome, Centenarians

## Abstract

This review explores the role and health impacts of probiotics, focusing specifically on *Bifidobacterium* spp. It highlights the functionalities that Bifidobacteria can provide, underscored by the historical evolution of definitions and technological advancements related to probiotics. By examining the association between Bifidobacteria and longevity, this review suggests new avenues for health enhancement. Highlighting case studies of centenarians, it presents examples related to human aging, illuminating the potential links to longevity through research on *Bifidobacterium* strains found in centenarians. This review not only emphasizes the importance of current research but also advocates for further investigation into the health benefits of Bifidobacteria, underlining the necessity for continuous study in the nutraceutical field.

## Introduction

The concept of probiotics, stemming from the Greek phrase meaning “for life,” has undergone a remarkable evolution since its early inception (Kumar et al., 2020). From the initial definition of probiotics as substances secreted by one microorganism to stimulate the growth of another, the understanding of probiotics has significantly expanded and deepened over the decades (Day et al., 2019).The progression of definitions reveals an intricate journey from recognizing mere tissue extracts that stimulate microbial growth to identifying specific live microbial cultures or cultured dairy products that offer substantial health and nutritional benefits to the host (Marchesi and Ravel, 2015) (Table 1).

**Table 1:** Chronological overview of probiotics definitions and their evolution (Adopted and modified from Markowiak and Śliżewska (2018)

Year	Definitions	References
1965	A substance secreted by one microorganism which stimulates the growth of another	Lilly and Stillwell (1965)
1971	Tissue extracts which stimulate microbial growth	Sperti (1971)
1974	Organisms and substances that contribute to intestinal microbial balance	(Parker, 1974)
1989	Live microbial feed supplement which beneficially affects the host animal by improving microbial balance	Fuller (1989)
1992	Viable mono- or mixed culture of live microorganisms which, applied to animals or man, have a beneficial effect on the host by improving the properties of the indigenous microflora	Havenaar et al. (1992)
1996	A live microbial culture or cultured dairy product that beneficially influences the health and nutrition of the host	Salminen (1996)
1996	Living microorganisms which, upon ingestion in certain numbers, exert health benefits beyond inherent basic nutrition	Schaafsma (1996)
1998	Living microorganisms that on ingestion in certain numbers exert health benefits beyond inherent basic nutrition	Guarner and Schaafsma (1998)
1999	A microbial dietary adjuvant that beneficially affects the host physiology by modulating mucosal and systemic immunity, as well as improving nutritional and microbial balance in the intestinal tract	Naidu et al. (1999)
2001	A preparation of or a product containing viable, defined microorganisms in sufficient numbers, which alter the microflora (by implantation or colonization) in a compartment of the host and by that exert beneficial health effect in this host	Schrezenmeir and de Vrese (2001)
2002	Live strains of strictly selected microorganisms which, when administered in adequate amounts, confer a health benefit on the host	Food Agriculture Organization (2002)
2004	Preparation of viable microorganisms that is consumed by humans or other animals with the aim of inducing beneficial effects by qualitatively or quantitatively influencing their gut microflora and/or modifying their immune status	Fuller (2004)
2009	Live microorganisms, which when administered in adequate amounts, confer a health benefit on the host	Food Agriculture Organization (2002)
2013	Live strains of strictly selected microorganisms which, when administered in adequate amounts, confer a health benefit on the host	Hill et al. (2014)

By 1989, probiotics were recognized as live microbial feed supplements capable of beneficially altering the host’s microbial balance, marking a pivotal shift toward appreciating their role in enhancing the host’s wellbeing (Fuller, 1989). The definitions continued to evolve, emphasizing the viability and beneficial effects of mono- or mixed cultures of microorganisms on the host’s indigenous microflora (Havenaar et al., 1992). Importantly, the emphasis began to shift towards recognizing the positive health impacts beyond mere nutritional value, including enhancing both mucosal and systemic immune responses and promoting the nutritional and microbial harmony in the digestive system (Salminen, 1996; Schaafsma, 1996).

**Fig. 1:**
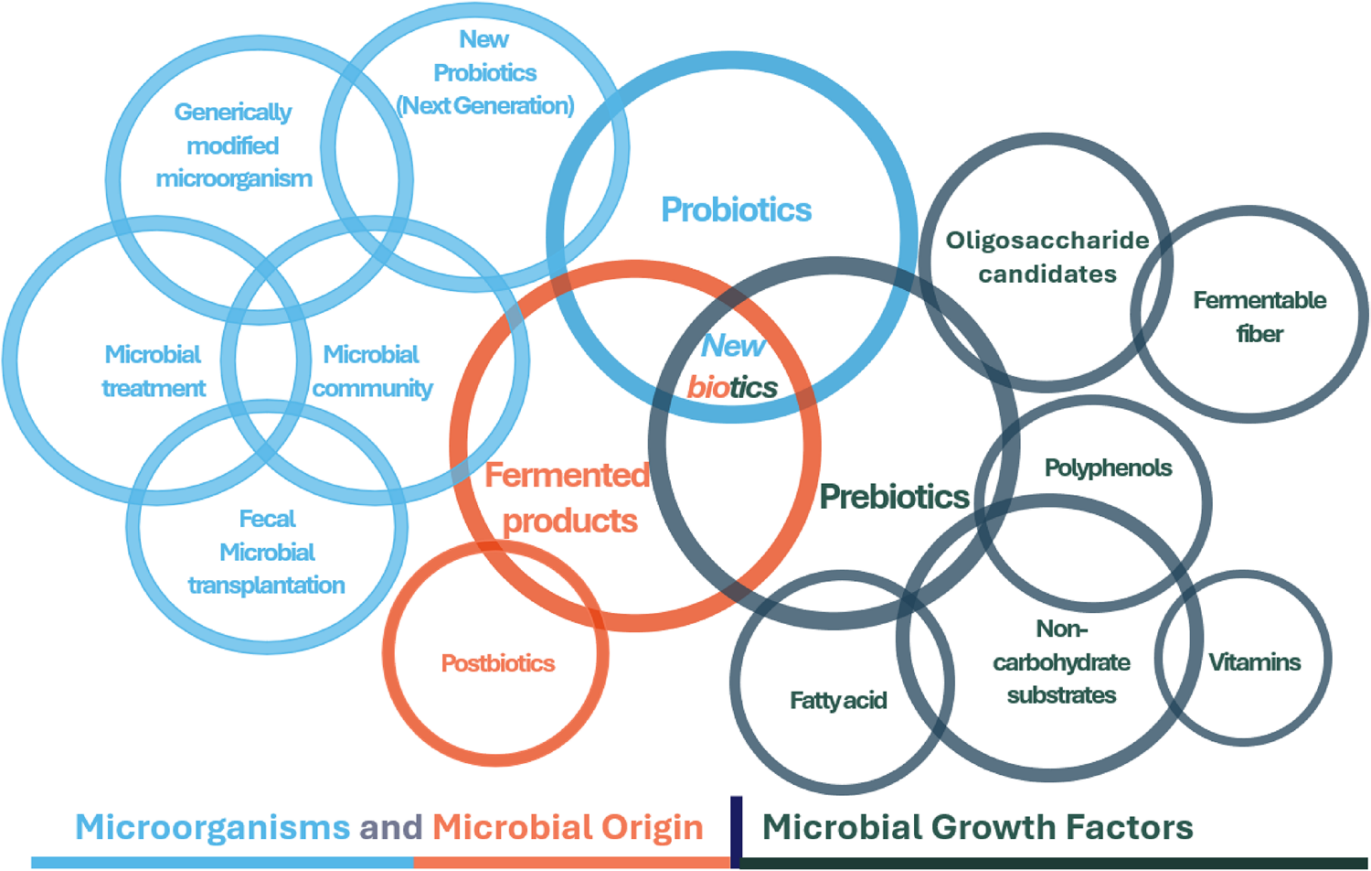
Mapping of probiotics, prebiotics, and postbiotics. The figure maps the interplay of pro-, pre-, and postbiotics within adjacent industry fields, underlining their extensive influence on health and industry progression (adopted and redesigned from Cunningham et al. (2021))

The turn of the millennium saw the development of a more refined understanding, highlighting preparations or products containing viable, defined microorganisms that exert health benefits by altering the microflora in a compartment of the host (Guarner and Schaafsma, 1998; Naidu et al., 1999; Schrezenmeir and de Vrese, 2001). By 2002, the definition honed in on the necessity of administering live, strictly selected microorganisms in adequate amounts to confer health benefits, thereby underscoring the precision and specificity required in probiotic formulations (Food Agriculture Organization, 2002; Fuller, 2004). Recent definitions, such as those from 2009 and 2013, encapsulate the cumulative knowledge and insights gained over years of research, affirming that live microorganisms, when administered in appropriate amounts, confer a health benefit on the host (Hill et al., 2014; Sanders, 2009). This evolution in the conceptualization of probiotics mirrors the burgeoning research in this field and reflects a deepening understanding of the complex interactions between microorganisms and their hosts. It underscores the continuous exploration and recognition of the potential of probiotics in promoting health and well-being and highlights the dynamic and ever-expanding frontier of probiotic research (Fig. 1).

As awareness of health among food consumers increases, the nutraceutical market has seen the introduction of new health functional foods (POLARIS_Market_Research, 2022; Size, 2022). Among these, probiotics have received significant attention for their functionality (POLARIS_Market_Research, 2022). The beneficial effects of probiotics are largely derived from their diverse interactions with the host’s intestinal microbiome, as detailed by Raheem et al. (2021). These interactions lead to several positive outcomes, such as the fortification of the intestinal barrier by adjusting the levels of proinflammatory cytokines and chemokines; enhancing the specificity of the intestinal barrier through the bolstered production of mucin, immunoglobulin A (IgA), and defensins; and bolstering the intestinal epithelial layer’s strength through increased synthesis of essential nutrients like vitamins, minerals, short-chain fatty acids (SCFAs), and growth regulators. Furthermore, probiotics aid in the production of substances that inhibit angiogenesis and increase the levels of beneficial cytokines (IL-2 and IL-12) and antioxidants, contributing to a decrease in intestinal pH and an upsurge in anti-inflammatory compounds, thereby boosting immune health (Tegegne and Kebede, 2022). They also play a crucial role in altering the composition of the gut microbiota, guiding the processes of cell apoptosis and differentiation, and inhibiting harmful pathways such as tyrosine kinase (Yadav et al., 2022).

Ongoing reports on the clinical and preclinical efficacies of various probiotic microbial strains have driven the global probiotic market to surpass $61 billion by 2021 (POLARIS_Market_Research, 2022). Recent studies have further highlighted the potential of probiotics in several key areas, including improving intestinal function, offering anti-allergic actions in the immune system (Liu et al., 2022), providing antibacterial effects against *Helicobacter pylori* strains (Acharjee et al., 2022; Liu et al., 2022), preventing autoimmune diseases (Liu et al., 2018), suppressing metabolic disorders (Li et al., 2021), and even suggesting potential anti-cancer effects (Lu et al., 2021). Through these multifaceted actions, probiotics demonstrate a comprehensive approach to enhancing host health, underlining their significance in the realm of health-functional foods.

**Fig. 2:**
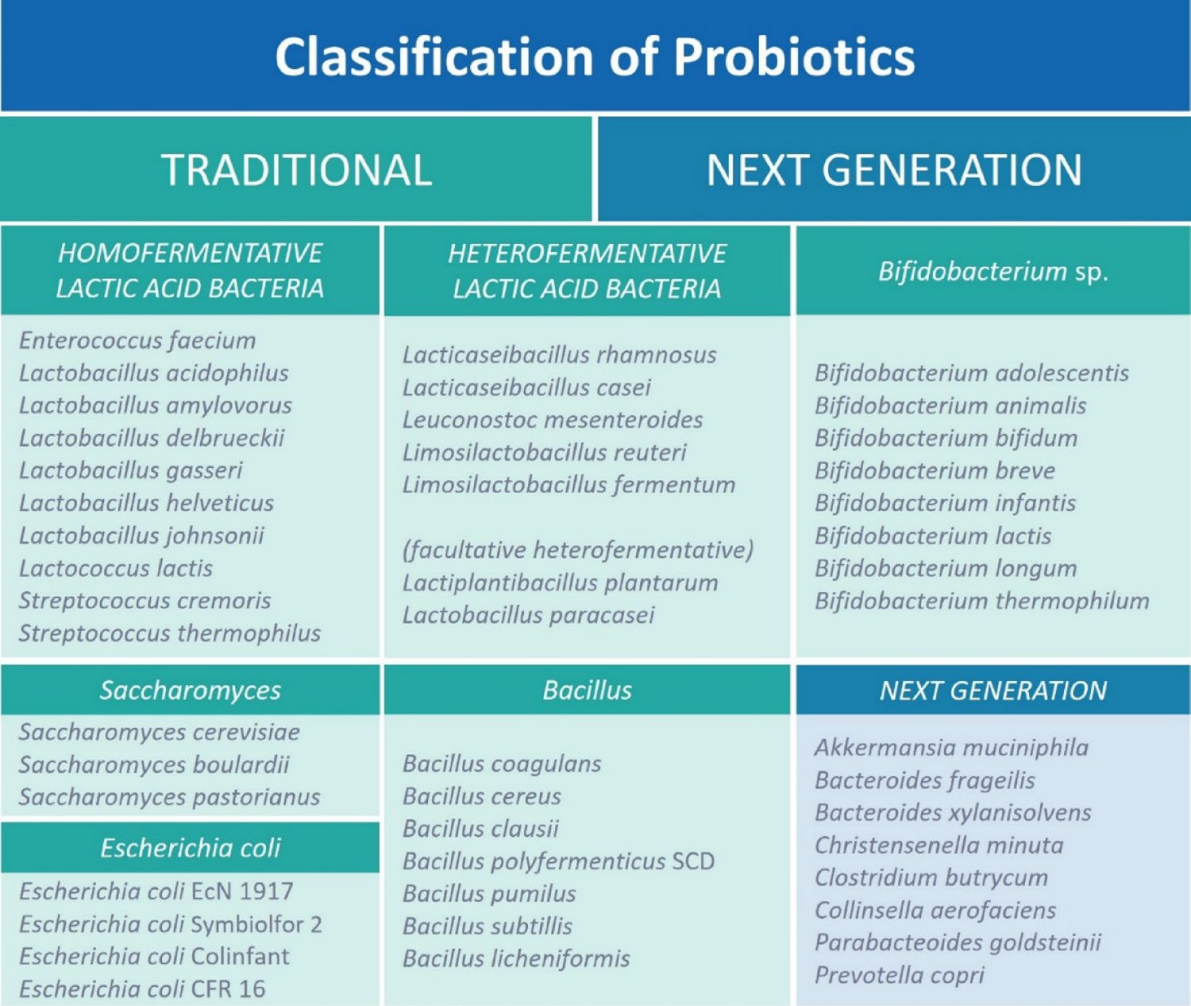
Probiotics classification (Compiled and updated based on information from Gattupalli and Gattupalli (2021); Lee et al. (2019), Sharma et al. (2014), Singh and Natraj (2021), Średnicka et al. (2021), Suez et al. (2020))

Probiotics are primarily composed of gram-positive bacteria, including the species of *Lactobacillus* and *Bifidobacterium* (Abdelhamid et al., 2019). Other nonpathogenic species with probiotic properties that have undergone commercial development include certain strains of *E. coli, Enterococcus, Pediococcus, Streptococcus, Lactococcus,* fungal strains (e.g., *Aspergillus oryzae*), and yeasts (e.g., *Saccharomyces boulardii*). Traditional probiotics composed of lactic acid bacteria, *Bifidobacterium* spp., *Saccharomyces*, *E. coli*, and *Bacillus* are commonly isolated from fermented foods, breast milk, and gut and generally recognized on the strain level. On the other hand, next generation probiotics (NGP) is mostly come from commensals and strict safety criteria and regulation is applied to NGP. Also, NGP may contain genetically modified organisms targeting specific diseases. (Singh and Natraj, 2021) (Fig. 2).

*Streptococcus oralis* and *S. salivarius*, contribute to health advantages (Bidossi et al., 2018; Burleigh et al., 2023; Burton et al., 2006). Recent research consistently underlines the crucial interactions between consumed probiotics and the host’s endemic microbiota. These interactions have been shown to bolster both the body’s natural and learned immune mechanisms, underscoring the probiotics’ capabilities to fend off pathogens and mitigate inflammation. Additionally, probiotics have been found to improve the absorption and availability of particular naturally occurring or metabolically produced compounds and vital nutrients, simultaneously alleviating food sensitivities in prone individuals (Michael and Joseph, 2020). Consequently, probiotics, similar to the gut’s native microbiota, can beneficially affect the overall well-being of the host by enhancing digestive health and immune function.

The molecular mechanisms of action of the compounds produced by probiotic microbes vary due to their diverse nature and bioactive organic composition (Suez et al., 2019). This variety enables beneficial effects through several mechanisms, including facilitation of direct cell-to-cell contact in the gut through various cross-feeding mechanisms and secretion of multiple molecules into the intestinal environment (Ma et al., 2023). These secretions play crucial roles in the complex interactions between gut microbiota, intestinal immunity, and epithelial cells. The molecular mechanisms mainly involve different types of proteins (located on or secreted by microbial surfaces), low-molecular-weight peptides and/or amino acids, bacterial DNA, and especially SCFAs (Cunningham et al., 2021; Ma et al., 2023). Like bacterial cell surface fragments, probiotic antigens can cross the intestinal barrier and stimulate the immune system, thereby impacting various physiological functions of the host.

Unlike traditional supplements, which typically target specific parts of the body, probiotic products are regarded as reliable assets that confer benefits across multiple body areas. The beneficial effects of ingested probiotics in improving the host’s immune system have been evident in treating disorders ranging from allergies and diarrhea to inflammatory bowel disease (IBD), irritable bowel syndrome (IBS), infections, infant colic, and even certain types of cancer (Casen et al., 2015; Selvamani et al., 2022; Zhou et al., 2022). This has led to the discovery of probiotic bacterial strains that mitigate the side effects of antibiotic treatments while enhancing their effectiveness. These strains can provide added benefits by reestablishing a healthy gut microbiota in patients undergoing extensive antibiotic treatment.

Advancements in next-generation sequencing (NGS) technology have further revolutionized microbial research, food and pharmaceutical quality control, and safety inspections (Jagadeesan et al., 2019). Many organizations now recommend using NGS technology to monitor the presence of microbial species in health-functional products (Suez et al., 2020). This not only aids in elucidating the functionality of probiotic products, but it also strengthens quality control. In the United States, approximately 3.9 million adults consume probiotic products annually (Parker et al., 2018). Moreover, sixty-four percent of those who take probiotics daily do so without a recommendation from a healthcare provider (Ozdener-Poyraz et al., 2022).

In Korea, the probiotics market has already exceeded 834 billion won, making probiotics the second most popular product in the health supplement market, second only to red ginseng (BIOTIMES, 2024). The Asia–Pacific region, in particular, is projected to grow at a CAGR of 9.6% by 2028 due to increased health awareness and improved consumption levels (Mordor_Intelligence, 2024). This market growth is attributed to strengthened scientific evidence confirming probiotic efficacy, increased consumer awareness, and a rising interest in health, especially in Korea, where rapid aging and preparation for a healthy old age are expected to expand the market (Kwon, 2023). These developments underline the importance of ensuring that health supplement producers provide accurate information and consultation to consumers.

Despite the market’s positive response, the majority of products in the current probiotics market consist predominantly of lactobacilli. By contrast, products that primarily contain Bifidobacteria as the active strain are rare. This rarity is not due to an inferior functionality of Bifidobacteria compared to lactobacilli; rather, it reflects the sensitivity of Bifidobacteria, as obligate anaerobes, to environmental challenges (Shigwedha and Jia, 2013). This sensitivity makes these microbes unsuitable for cultivation, distribution, and production in present commercialization processes. Delivering viable Bifidobacteria in food products is particularly challenging due to the rigors of food processing and storage, which include exposure to low pH, oxygen, heat, and cold (Ruiz et al., 2011). This distinction underscores the complexities and difficulties in leveraging the benefits of *Bifidobacterium* strains through direct supplementation in food products, leading to a commercial preference for *Lactobacillus*-based product production and marketing.

In the mammalian gut, selectively anaerobic microorganisms, such as lactobacilli, enterococci, and intestinal bacteria, begin to form colonies immediately after birth. Following the initial establishment of colonies by selectively anaerobic microbes such as lactobacilli, enterococci, and other gut bacteria after birth, there is a progressive colonization by obligate anaerobes including species of *Bifidobacterium, Bacteroides*, and *Clostridium*. This process leads to a gradual shift from a higher prevalence of facultative anaerobes to a dominance of obligate anaerobes over time (Krupa-Kotara et al., 2023; Tanaka and Nakayama, 2017). Bifidobacteria and lactobacilli, vital to the gut flora of many mammals, emerge as the most thoroughly studied and extensively marketed probiotics due to their critical roles in the intestinal ecosystem (Encyclopedia of Dairy Sciences 2011). Although both groups are characterized as non-spore-forming, gram-positive bacteria that produce lactic acid, their metabolic pathways diverge significantly. Lactobacilli primarily metabolize refined sugars into lactic acid, showcasing limited biosynthetic abilities, whereas Bifidobacteria play a crucial role in generating SCFAs, highlighting a distinct metabolic capability (Vlasova et al., 2016). Although they share some common characteristics, the lactobacilli and Bifidobacteria belong to very different phylogenetic groups: lactobacilli to the Firmicutes phylum and Bifidobacteria to the Actinobacteria phylum (Vlasova et al., 2016). Therefore, lactobacilli and Bifidobacteria can be anticipated to exhibit many differences in their activities and characteristics within the host body.

Providing information on the health functionality of Bifidobacteria therefore represents an important responsibility for the academic community to recognize. In this context, this review aims to offer a new perspective on *Bifidobacterium* spp. as vital probiotics. The review delves into the clinical implications and promising prospects associated with *Bifidobacterium* strains, particularly in light of the intriguing relationship now identified in the gut microbiomes of centenarians. By exploring the unique composition of Bifidobacteria in centenarians and the potential implications for longevity, the review seeks to uncover valuable insights into approaches for harnessing the therapeutic benefits of Bifidobacteria as dietary supplements.

## Bifidobacteria and the centenarian microbiome

### Italy

The composition of the gut microbiota is widely recognized to change in tandem with human aging (Nagpal et al., 2018). Recent studies employing molecular biology techniques have revealed clear differences in the gut microbiota composition across various age groups, including infants, toddlers, adults, and the elderly (Yan et al., 2022). Many researchers have highlighted the presence of Bifidobacteria as a biological marker for a “healthy” intestinal state in humans, with their probiotic function being well documented (Chen et al., 2021). While numerous studies have noted a decrease in both the count and diversity of *Bifidobacterium* strains within the gut microbiota of the broader elderly population (Arboleya et al., 2016), ongoing research has recorded a higher proportion of Bifidobacteria among centenarians—those over 100 years of age—compared with their younger elderly counterparts residing in the same region (Arboleya et al., 2016; Drago et al., 2012; Kato et al., 2017; Luan et al., 2020; Odamaki et al., 2016; Sepp et al., 2022; Wang et al., 2015). This observation suggests that the longevity of centenarians might be linked to distinct characteristics within their gut microbiota. Increases in certain essential gut bacterial species have been reported in the microbiota of centenarians, whereas others show a significant decline (Arboleya et al., 2016; Drago et al., 2012; Kato et al., 2017; Luan et al., 2020; Odamaki et al., 2016; Sepp et al., 2022; Wang et al., 2015). The microbiota composition in centenarians is likely influenced by an optimal mix of genetics, environmental conditions, diet, and lifestyle choices (Strasser et al., 2021); however, discerning the patterns in the gut microbiota of centenarians can provide food microbiologists with valuable insights into promising probiotics. For example, the development of probiotics that are modeled on the predominant microbiome compositions found among centenarians could serve as an effective strategy for addressing diseases such as obesity and diabetes and for extending the overall human lifespan (Biagi et al., 2016; Rampelli et al., 2020). Although the precise identification or mimicry of the factors contributing to the longevity of centenarians may not be possible, introducing key bacterial strains as probiotics or incorporating them through diet could represent an initial step toward prolonging an individual’s lifespan.

The research team led by Kelvin and Carru (Wu et al., 2019) has conducted a thorough investigation into the gut microbiota composition and functional capabilities of centenarians living on the island of Sardinia, Italy, and has shared their findings through microbiome analysis data. Notably, Sardinia has a high proportion of residents living beyond 100 years, making it a focal point for studies aimed at unlocking the secrets of longevity. Located in the Mediterranean, Sardinia’s isolated environment may contain unique elements that contribute to its inhabitants’ long lives, although the precise reasons remain to be clarified. In this context, the research group has suggested that the Sardinian centenarians’ gut microbiota might provide crucial insights into achieving longevity.

Employing metagenomics sequencing, the team investigated the compositional and functional disparities in the gut microbiota across different age groups within the Sardinian populace. The results showed that the microbiome profiles of the island’s younger, healthy residents were similar to those of the general elderly population. By contrast, the centenarians had a distinct gut microbiota composition that differed from both younger and other elderly groups, suggesting a potential relevance to healthy aging and longevity. The study particularly highlighted the importance of *Bifidobacterium* strains, with a focus on *Bifidobacterium adolescentis*, a species known for its significant influence on the production of Th17 cells that manage intestinal inflammatory responses and immune regulation. A notable discovery was the reduction of certain bacteria (*Faecalibacterium prausnitzii* and *Eubacterium rectale*) and a higher presence of *Methanobrevibacter smithii* and *Bifidobacterium adolescentis* in centenarians’ intestines. Despite the diminished carbohydrate degradation abilities of these species, these findings hint at a shift toward a microbial composition that supports key metabolic processes, such as glycolysis and fermentation to produce SCFAs, as suggested by the microbiome analysis. The group proposes that positive alterations in gut microbiota, as indicated by the increased prevalence of beneficial bacteria such as *Bifidobacterium adolescentis*, could positively affect the metabolic environment of hosts aged over 100 years, possibly by boosting the production of advantageous metabolites, such as SCFAs. An important point to note is that *Lactobacillus* species, which are commonly used as probiotics, were not included in the top 10 genera (*Escherichia, Streptococcus, Roseburia, Subdoligranulum, Alistipes, Ruminococcus, Faecalibacterium, Bifidobacterium, Eubacterium,* and *Bacteroides*) identified by this research group. This suggests that *Bifidobacterium* species may play a significant functional role as probiotics that contribute to longevity in hosts.

These findings align with those of a study conducted by Biagi and Candela research group (Biagi et al., 2016) in Emilia Romagna, another region of Italy. According to their research, Bifidobacteria were more abundant in the feces of centenarians than in elderly individuals. Specifically, the prevalence of *Bifidobacterium* spp. in the semi-supercentenarian group aged 105 years and older was reported as 92%, the highest among the studied groups. The prevalence of *Bifidobacterium* spp. in the group aged 99–104 years (centenarian group) was 87% and significantly higher than the 80% observed in the general elderly group aged 65–75 years. These results suggest a potential link between the gut microbiota composition and the health and longevity of semi-supercentenarians and centenarians. Therefore, the specific composition of gut microbiota—and particularly the abundance of Bifidobacteria—in the semi-supercentenarian group can be postulated to contribute to healthy aging and longevity.

### Estonia

A human clinical study by Mändar group (Sepp et al., 2022) reported a comparative analysis of the gut microbiome compositions of centenarians, noted for their maintained cognitive functions, and younger cohorts, with the aim of exploring the influences of childhood living conditions and dietary habits on microbiome development and composition in longevity achievers. Employing NGS techniques, this research group focused on examining the diversity and composition of gut microbiota, including *Bifidobacterium* spp. The study uncovered a significant elevation in the relative abundance of specific Bifidobacteria, notably *Bifidobacterium dentium* and *Bifidobacterium longum*, in the feces of Estonian centenarians compared to younger individuals (p = 0.012 and p = 0.041, respectively). This finding is particularly significant, given the known beneficial effects of *Bifidobacterium* spp. on gut health and immune function.

Previous studies have highlighted the unique ability of *Bifidobacterium dentium* to boost mucin synthesis and secretion, despite its lack of enzymes for mucin glycan degradation. This capability suggests a potential for increasing mucin production through upregulation of the expression of genes, such as *MUC2,* and induction of autophagy signaling pathways. Furthermore, bioactive molecules produced by *B. dentium*, like γ-aminobutyric acid (GABA), are believed to stimulate calcium signaling and MUC2 release, thereby facilitating autophagy. Similarly, acetate from *B. dentium* has been shown to elevate MUC2 levels in T84 cells. *B. longum* is well regarded for its capacity to bolster immune function, mitigate inflammatory responses, and ameliorate the gut environment, thereby offering prospects for supporting healthy aging and longevity. Recent controlled trials in elderly populations have demonstrated the ability of *B. longum* to mitigate symptoms of chronic constipation and reduce perceived psychological stress.

An investigation conducted by Mändar research group (Sepp et al., 2022) on centenarians further illuminates the profound connection between dietary habits and the microbiome. The dietary preference of the longevity group for potatoes and cereal products over the food choices of the younger group implies that the centenarian food choices may supply the fiber and nutrients favorable for Bifidobacterial growth. This study accentuates the complex interplay between diet, the gut microbiome, and longevity, and provides valuable insights for augmenting the healthspan and lifespan through targeted dietary and microbial interventions.

### Japan

A comprehensive study conducted in 2017 by the Kato group (Kato et al., 2017) meticulously investigated the dynamics of *Bifidobacterium* species composition across a broad age range, from newborns to centenarians. The aim of this research, involving 441 healthy Japanese participants, was to elucidate the complex dynamics and potential symbiotic relationships among *Bifidobacterium* spp. that remain closely associated with humans throughout the human lifespan. The findings consistently identified *B. longum* as the predominant species across all age groups, with the highest detection rate among the studied species at 88.1%. This highlights the critical role of *B. longum* within the human gut microbiota throughout an individual’s lifespan, underscoring its importance in maintaining gut health from infancy through advanced age. While *B. catenulatum* and *B. bifidum* were also commonly detected across almost all age groups, excluding centenarians, they showed lower detection rates of 61.7% and 28.3%, respectively, when compared to the *B. longum* group. Notably, the *B. catenulatum* and *B. adolescentis* groups became dominant after weaning.

The widespread distribution and high detection rate of the *B. longum* group emphasize its significance as an integral component of the gut microbiota from infancy through old age, reaffirming its pivotal role at various stages of human life. *Bifidobacterium breve* was notably present in children under the age of three (detected in 71.4% of the subjects) and maintained high detection rates in individuals under 10 years of age. This indicates a crucial role for *B. breve* in the gut microbiota during early childhood. The detection rate of *B. dentium* varied with age, as it was identified in individuals over age 20 and increased in prevalence up to 90 years of age, suggesting a specific role for *B. dentium* within the microbiota of older adults. By contrast, *B. animalis* ssp. *lactis*, which is not considered a typical component of the human gut microbiota, was detected in a limited manner in 11.4% of the subjects, primarily among individuals from weaning to under 80 years of age. Low detection rates were also observed for the *B. gallinarum* group, which were detected only in individuals aged between 31 and 84 years, while *B. minimum* and *B. mongoliense* were not detected at any age. The correlation among *Bifidobacterium* species revealed a significant association between the presence of the *B. longum* group and the presence of the *B. catenulatum* group, *B. breve*, the *B. adolescentis* group, *B. bifidum*, and the *B. gallinarum* group. By contrast, *B. animalis* ssp. *lactis* did not show a significant correlation with other *Bifidobacterium* species.

This investigation offers profound insights into the changes and significance of *Bifidobacterium* species throughout the human life cycle. The enduring presence and high detection rate of *B. longum* from newborns to those over 100 years of age underscore its central role within the human gut microbiota and support its essentiality for maintaining a healthy gut environment. These findings provide valuable information for optimizing the gut microbiota in relation to age and health status, while illustrating the specific patterns of dominant *Bifidobacterium* species at different life stages.

### China

Zhao and Li research group (Wang et al., 2015) used next-generation sequencing analysis to compare the gut composition of *Bifidobacterium* species in the intestines of centenarian residents and younger elderly in Bama County, Guangxi Province, China. Specifically, this study involved participants from different age groups and locations. The centenarians (Group B; n = 8) were aged 100 to 108 years, while the younger elderly (Group M, n = 8) were aged 80 to 99 years. An additional group of younger elderly aged 80 to 99 years (Group A) was recruited from Nanning, the provincial capital city. The researchers used PCR-DGGE, clone libraries, and qPCR to analyze the composition of *Bifidobacterium* species in the guts of the participants. The results revealed that the *Bifidobacterium* microbiota in centenarians differed notably from that of younger elders (aged 80 to 99), with centenarians exhibiting a slightly higher diversity in fecal *Bifidobacterium* species compared to their younger counterparts.

The study identified eight different *Bifidobacterium* species. *B. dentium* and *B. longum* were predominant in the gut microbiota of both centenarians and younger elders, whereas *B. minimum*, *B. saecularmay/B. pullorum*/*B. gallinarum*, and *B. mongoliense* were exclusively found in centenarians. Centenarians tended to harbor a more diverse range of fecal *Bifidobacterium* species compared to younger elders from other regions. Genomic analysis of *B. dentium* revealed that approximately 14% of its genes are associated with carbohydrate metabolism and transport proteins, indicating significant genetic adaptation to complex carbohydrate metabolism. *B. thermophilum*, primarily known as a commensal species associated with animals but also detected in the human gut, emerged as the second most prevalent *Bifidobacterium* species*.* The fourth most prevalent *Bifidobacterium* species in centenarians from Bama was *B. pseudocatenulatum*/*B. catenulatum*, suggesting potential differences in beneficial effects compared to other populations. The frequency of *B. adolescentis* was lower in centenarians and younger elders from Bama, indicating its greater susceptibility to acidic and oxidative stresses, which may hinder its colonization in acidic environments induced by *B. dentium* proliferation. Several clones, including *B. mongoliense*, *B. minimum*, and *B. saecularmay/B. pullorum*/*B. gallinarum*, were also detected in the clone libraries of centenarians, but not in younger elders, hinting at potential dietary influences on species presence.

In summary, the study revealed differences in the composition of fecal *Bifidobacterium* species between centenarians and younger elders, with centenarians showing slightly higher diversity. The investigation by the Zhao and Li group sheds light on the unique composition of fecal *Bifidobacterium* species in centenarians and underscores their potential role in longevity. These findings suggest that centenarians harbor a distinct array of *Bifidobacterium* species in their gut microbiota compared to younger elders, emphasizing the intricate interplay between gut microbiota and aging processes. However, acknowledging the limitations imposed by the small sample size of only 8 individuals per group is essential, as this creates constraints on sample size, potential lack of diversity, and susceptibility to external influences.

## Recommendations for consumer health and the probiotics industry

Probiotics are essential to human health, as their physiological activities within the gastrointestinal tract are crucial for improving digestion and bolstering the immune system (Ashaolu, 2020). Nevertheless, to unleash their full potential, probiotics must be able to endure the gastrointestinal tract’s stringent conditions, such as those created by stomach acid and bile, and successfully colonize the intestines (González-Rodríguez et al., 2013). For this reason, innovative coating and encapsulation technologies have been developed for commercial probiotic preparations to significantly enhance probiotic intestinal colonization (Pop et al., 2019). The Bifidobacteria are particularly effective as colon residents, as they provide several benefits, including boosting the colonic immune system, suppressing pathogenic bacteria, and facilitating digestion. However, despite the predominance of live culture–based products in the current probiotic market, the Bifidobacteria, as obligate anaerobes, often exhibit low survival rates when navigating the gastrointestinal tract (Venema et al., 2019). Thus, the creation of novel *Bifidobacterium* strains to improve the intestinal colonization by beneficial Bifidobacteria is of paramount importance (Colston et al., 2022).

Encouraging findings from diverse studies have highlighted high intestinal colonization rates for Bifidobacteria. Investigations using culturing methods across the human lifespan have shown that *Bifidobacterium* and *Lactobacillus* species coexist within the human gut. However, research by Mitsuoka’s group (Mitsuoka (2014), Fig. 3) indicates that higher intestinal colonization is achieved by Bifidobacteria than by lactobacilli throughout life.

**Fig. 3:**
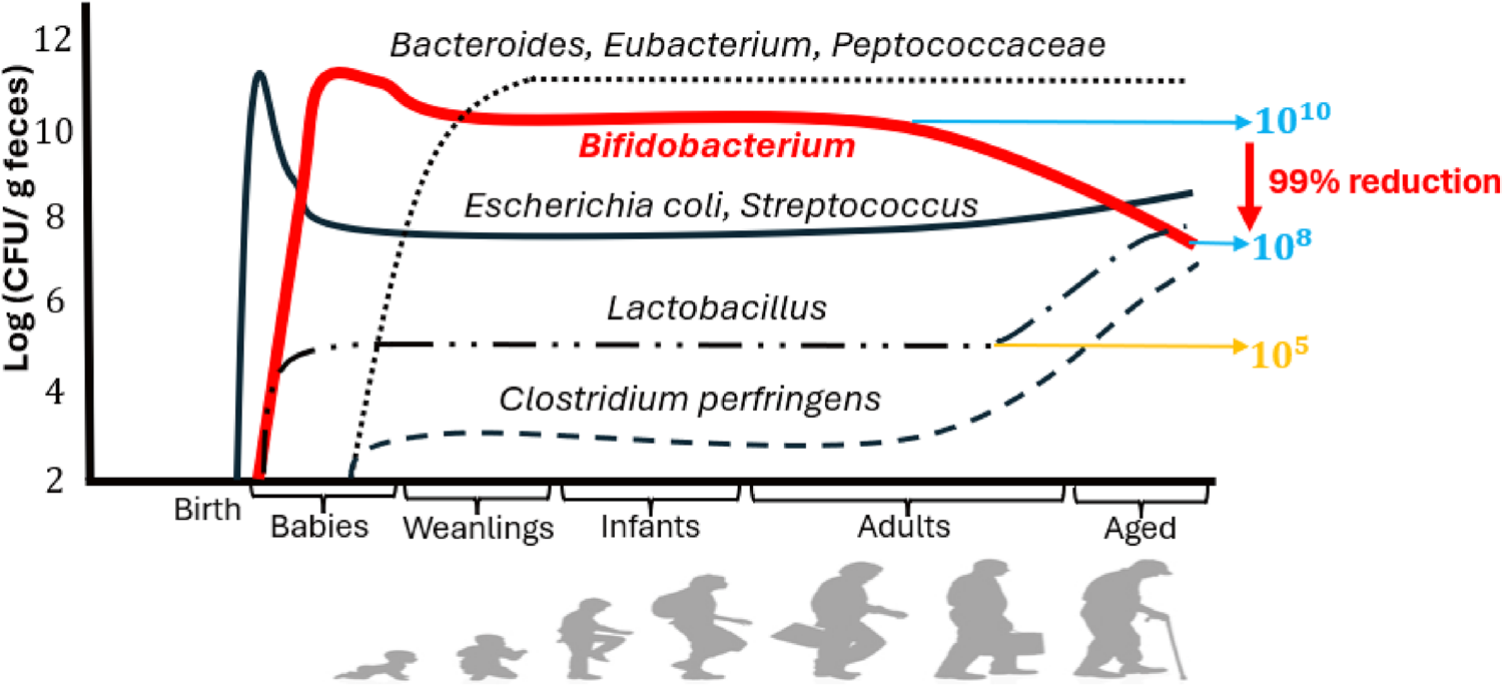
Changes in gut microbial populations and dynamics of *Bifidobacterium* levels across the human lifespan (adopted and modified from Arboleya et al. (2016), Mitsuoka (2014))

Bifidobacterial levels in feces, at around 11 log CFU/g in infancy, decrease by roughly 90% to 10 log CFU/g in adulthood, and plummet further to 9 ~ 8 log CFU/g in old age, for a reduction of 99%. Interestingly, the concentrations of lactobacilli, maintained at about 5 log/g from infancy through middle age, increase as age progresses.

A study by the Sung group in 2020 (Gupta et al., 2020) on the Gut Microbiome Health Index sheds light on 50 microbial species integral to a healthy gut ecosystem. Of these, seven were designated as Health-prevalent species, with the remainder classified as Health-scarce species. Remarkably, within the Health-prevalent species, three were *Bifidobacterium* species (*Bifidobacterium adolescentis*, *Bifidobacterium angulatum*, and *Bifidobacterium catenulatum*). Moreover, among the 50 species associated with a healthy gut ecosystem, only one *Lactobacillus* species (*Lactobacillus salivarius*) was mentioned and was deemed a Health-scarce species due to a 10.5-fold difference in prevalence between healthy and nonhealthy samples.

In 2020, the Hall group conducted (Alcon-Giner et al., 2020) a study involving 234 infants, demonstrating that dietary supplementation with *Bifidobacterium* and *Lactobacillus* species resulted in fecal 16S rRNA gene profiling data predominantly showing *Bifidobacterium*. Notably, the microbial community of the Bif/Lacto group exhibited a higher relative abundance compared to the control group at all time points, suggesting that the supplemented strains could persist in the infant’s microbiota. This persistence indicates the potential for promoting colonization by other *Bifidobacterium* spp. Infants treated with both *Bifidobacterium* and *Lactobacillus* (Bif/Lacto group) also showed a lower relative abundance of other microbes, such as *Klebsiella, Escherichia,* and *Enterobacter* species, compared to the control group, suggesting that supplementation could replace potentially pathogenic microbes with a healthier microbial community composition. *Lactobacillus* species were detected in only a minority of infants; however, lactobacilli showed a higher relative abundance in the Bif/Lacto group than in the control group at all points, indicating that the persistence of lactobacilli may be transient and limited.

The high detection rates of *Bifidobacterium* and *Lactobacillus* species in infants, coupled with the low presence of *Klebsiella, Escherichia, Enterococcus,* and *Clostridium*, are noteworthy, as these features reveal that the relative abundance of lactobacilli is not significant in most infants. The dominance of Bifidobacteria in the early stages (0–9 days) started with higher Shannon diversity and inverse Simpson diversity values between the Bif/Lacto group and the control group of infants but decreased over time in the Bif/Lacto group compared to the control. This could be associated with the increased abundance of Bifidobacteria, suggesting that the dominant presence of *Bifidobacterium* in the immature microbiota might lead to lower diversity. Consequently, Bif/Lacto supplementation is related to the ability of *Bifidobacterium* species to establish a dominant presence in the immature intestinal microbiota, potentially inducing positive changes in the composition and function of the microbiota.

Similar results to those reported by Hall’s group were previously reported by the Ji group in 2003 (Kim et al., 2003). In their study, 4 g of *Bifidobacterium*-fermented soy hypocotyls (BFSH) containing 9 log cfu/g of Bifidobacteria were administered daily to 14 elderly individuals for 10 days, followed by a 10-day non-intake period, and repeating this cycle for a total of 50 days. They observed a significant increase in fecal *Bifidobacterium* species and a decrease in *Bacteroides* species during BFSH administration. The high relative abundance of Bifidobacteria could improve the intestinal environment and decrease pathogenic microbes, ultimately benefiting the health and development of the elderly.

In the development of probiotic *Bifidobacterium* strains, the creation of products containing bacteriocins or dead cells is also a crucial consideration (Siciliano et al., 2021). Compared to live cultures, these products offer ease of storage and transport and higher safety for use. Given the current trend toward stringent regulations on claims of efficacy against disease, the development of *Bifidobacterium* strains must adopt an evidence-based approach, including thorough research on strain identification, safety, and functionality, as well as verification of efficacy through clinical trials (Abdelhamid et al., 2019; Fijan, 2014). Despite hundreds of *Bifidobacterium* strains now in commercial use worldwide, only about 30 strains have received GRAS (Generally Recognized As Safe) certification from the FDA since 2005 (US_FDA, 2023) (Table 2).

**Table 2:** List of commercial *Bifidobacterium* cell strains granted GRAS certification by the FDA

*Bifidobacterium* cell strains	Notifier	Notifier country	Date of closure	GRN no
*B. breve* DSM 33444	Chr. Hansen A/S	Denmark	Jul 10, 2023	1114
*B. animalis* subsp. *lactis* IDCC 4301	Ildong Bioscience Co., Ltd	Republic of Korea	Jun 23, 2023	1092
*B. bifidum* strain NITE BP-31	Meiji Co., Ltd	Japan	Apr 5, 2023	1090
*B. lactis* strain KCTC 11904BP	Reims, Inc	United States	Oct 31, 2023	1082
*B. longum* subsp. *infantis* M-63	Morinaga Milk Industry Co., Ltd	Japan	Apr 26, 2022	1003
*B. breve* strain MCC1274	Morinaga Milk Industry Co., Ltd	Japan	Jul 22, 2022	1002
*B. longum* subsp. *infantis* strain ATCC SD 6720	Danisco, USA Inc	United States	Dec 21, 2021	985
*B. animalis* subsp. *lactis* strain AD011	BIFIDO Co., Ltd	Republic of Korea	Mar 17, 2021	952
*B. longum* subsp. *infantis* DSM 33361	Chr. Hansen, Inc	United States	Mar 1, 2021	950
*B. longum* BB536	Morinaga Milk Industry Co., Ltd	Japan	Dec 26, 2019	877
*B. animalis* subsp. *lactis* UABla-12	UAS Laboratories, LLC	United States	Dec 9, 2019	872
*B. animalis* subsp. *lactis* strain BB-12	Chr. Hansen, Inc	United States	Dec 9, 2019	856
*B. animalis* subsp. *lactis* strain R0421	Lallemand Health Solutions	Canada	Feb 5, 2020	855
*B. bifidum* BGN4	BIFICO CO., LTD	Republic of Korea	Jun 25, 2019	814
*B. longum* BORI	BIFICO CO., LTD	Republic of Korea	Jun 21, 2019	813
*B. longum* subsp. *infantis* R0033, and *B. bifidum* R0071	Lallemand Health Solutions	Canada	Aug 20, 2018	758
*B. breve* M-16 V	Danone Trading B. V	Netherlands	Sep 30, 2013	455
*B. breve* M-16 V	Morinaga Milk Industry Co., Ltd	Japan	Sep 27, 2013	454
*B. breve* M-16 V	Morinaga Milk Industry Co., Ltd	Japan	Sep 27, 2013	453
*B. animalis* subsp. *lactis* strains HN019, Bi-07, Bl-04 and B420	Danisco USA, Inc	United States	Apr 10, 2013	445
*B. animalis* subsp. *lactis* strain Bf-6	Cargill, Inc	United States	Sep 29, 2011	377
*B. longum* strain BB536	Morinaga Milk Industry Co., Ltd	Japan	Jul 8, 2009	268
*B. lactis* strain Bb12	Nestle USA	United States	Mar 19, 2002	49

At present, nine *Bifidobacterium* strains characterized by three Korean food and biotechnology companies, BIFIDO Co. Ltd., Cell Biotech Co. Ltd., and Ildong Bioscience Co., Ltd., have been put forward for FDA GRAS (Generally Recognized As Safe) certification. These strains include *Bifidobacterium lactis* KCTC 11904BP, *Bifidobacterium infantis* KCTC 11859BP, *Bifidobacterium breve* KCTC 12201BP, *Bifidobacterium longum* KCTC 12200BP, *Bifidobacterium bifidum* KCTC 12199BP, *Bifidobacterium animalis* subsp. *lactis* AD011, *Bifidobacterium bifidum* BGN4, *Bifidobacterium longum* BORI, and *Bifidobacterium animalis* subsp. *lactis* IDCC 4301. Among these, three strains from BIFIDO Co. Ltd., *Bifidobacterium animalis* subsp. *lactis* AD011, *Bifidobacterium bifidum* BGN4, and *Bifidobacterium longum* BORI, along with one strain from Ildong Bioscience Co., Ltd., *Bifidobacterium animalis* subsp. *lactis* IDCC 4301, have been officially evaluated for safety and have recently received GRAS certification (US_FDA, 2023).

Ultimately, the future of the probiotics market relies on the development of new *Bifidobacterium* strains with high intestinal colonization ability. This advancement will enable the production of probiotic products that provide higher intestinal survival rates and improved health effects, thereby increasing the likelihood of meeting the strict verifications required for health claims on probiotic products. Research and development efforts in this field must adopt a systematic approach based on scientific evidence.

## Probiotics and innovation

**Fig. 4:**
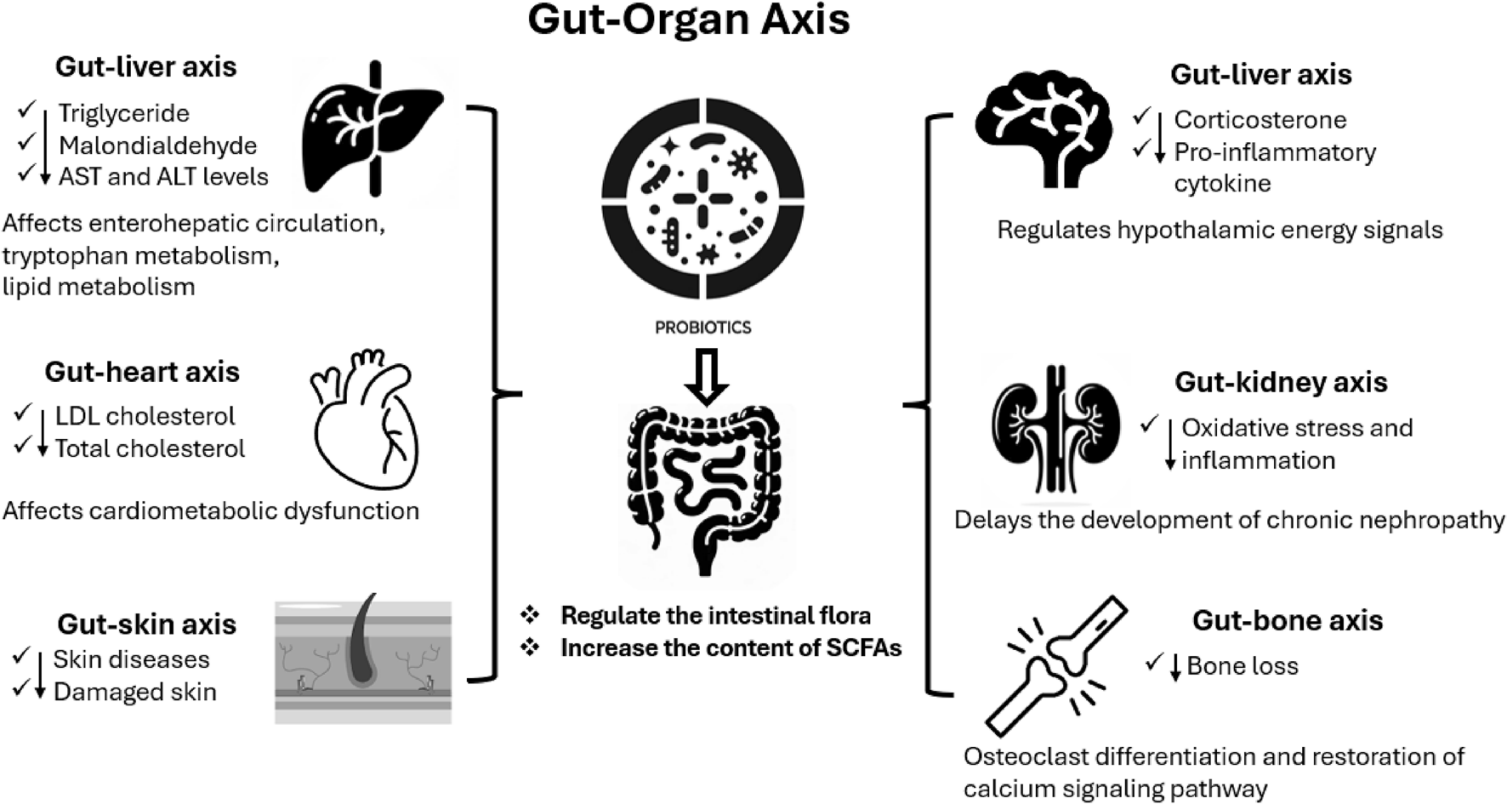
The schematic of the gut-organ axis illustrates the influence of the gut microbiome on systemic health, detailing the beneficial effects of probiotics on liver, heart, skin, brain, kidney, and bone health

Since Elie Metchnikoff’s pioneering discovery in 1907 (Anukam and Reid, 2007), the concept of probiotics has continually evolved, reflecting a deeper understanding of its impact on health and longevity. Recent studies on centenarians have particularly highlighted the positive effects of Bifidobacteria within the human gut microbiome, marking a significant advancement in the field (Wang et al. 2015; Kato et al. 2017; Sepp et al. 2022; Biagi et al. 2016; Wu et al. 2019). Showcasing its potential for continued research in the field of human longevity. The emergence of next-generation probiotics (NGPs) and live biotherapeutic products (LBPs) heralds a new era in restoring healthy states across diverse human ecosystems, including the gastrointestinal, hepatic, renal, skeletal, cardiac, cerebral, vaginal, and dermal systems. (Cordaillat-Simmons et al., 2020; Schemczssen-Graeff and Pileggi, 2022; Zhang et al., 2022) (Fig. 4).

**Fig. 5:**
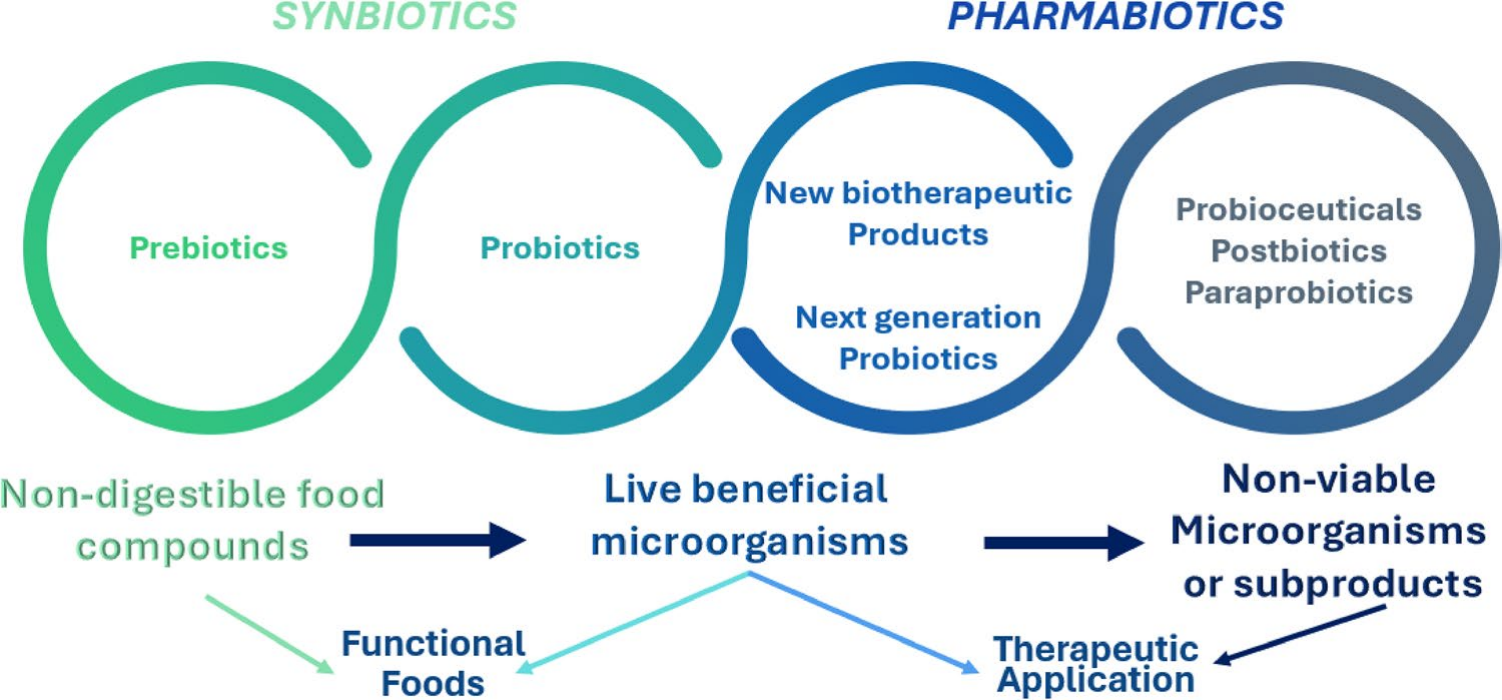
Evolutionary concepts in the functional probiotics-related industry domain

This development is poised to play a crucial role in enhancing the quality of life and health outcomes in aging societies. In addition, the introduction of varied terms, from traditional probiotics to prebiotics, synbiotics, pharmabiotics, postbiotics, paraprobiotics, and probioticals, reflects the exponential growth in knowledge within the probiotics research domain (LeBegue et al., 2020; Lin et al., 2019; Monteiro et al., 2023; Teame et al., 2020) (Fig. 5).

This is an effective multidisciplinary approach for the natural restoration of healthy states across different human ecosystems, which is crucial for improving elderly health and the quality of life in aging societies. Looking ahead, the probiotics industry and research domains face the challenge of leveraging the profound understanding of Bifidobacteria to maximize their beneficial impacts on human health. This involves the development of new products and technologies that ensure high survivability and beneficial effects of these microbes within the gut. These types of efforts require a systematic approach based on scientific evidence, highlighting the need for stringent standards and regulations to ensure product safety, efficacy, and quality (Cordaillat-Simmons et al., 2020). Consumers must be able to choose probiotic products that best suit their health needs, but this requires access to accurate information and consultations (Cordaillat-Simmons et al., 2020). Health supplement manufacturers bear the responsibility of providing reliable data based on scientific research aimed at continuously evaluating and enhancing the long-term health benefits of their probiotic products. The transformation of scientific discoveries into practical applications is pivotal in improving the quality of life for the aging population. Research uncovering the potential of Bifidobacteria in health enhancement and longevity, particularly through the exploration of specific strains found in the gut microbiomes of centenarians, offers invaluable insights. The resulting new knowledge supports the therapeutic benefits of regulating the gut microbiome with dietary supplements containing Bifidobacteria. Multiple research groups underscored the importance of developing probiotic strains that contribute to the well-being of the general population while also supporting healthy aging and extended lifespans, thereby emphasizing the need for ongoing research and development in this dynamic field.

## A new horizon

This review illuminates the pivotal role of probiotics, particularly *Bifidobacterium* species, and their positive impact on human health. Bifidobacteria aids in maintaining gut microbial balance, suppressing pathogenic microbes, enhancing digestion, and strengthening the immune system through various mechanisms (Ma et al., 2023). Research and technological advancements aimed at improving the intestinal colonization of these strains are vital for maximizing the potential of probiotics and providing tangible health benefits to consumers. This area of research offers profound insights into the diversity and functionality of *Bifidobacterium* species across different regions worldwide, spotlighting the characteristics of *Bifidobacterium* strains associated with longevity (Arboleya et al., 2016). These insights contribute to a better understanding of how Bifidobacteria interact with the human gut microbiota to promote health, while also providing essential guidelines for future probiotic product development. The future of the probiotics market relies heavily on the development of *Bifidobacterium* strains with a high ability for intestinal colonization. Advancements in this area will enable the creation of probiotic products that offer higher gut survival rates and enhanced health effects, thereby meeting the stringent verification required for health claims on probiotic products. A systematic approach based on scientific evidence is essential in probiotics research and development efforts (Khoruts et al., 2020; Ma et al., 2023). Importantly, strict standards and regulations are needed to ensure the safety, efficacy, and quality of probiotic products while prioritizing consumer health. Consumers should be able to choose the most suitable probiotic products for their health status and needs based on accurate information and consultation. Health supplement manufacturers must provide reliable data based on scientific research while also continuously assessing and improving the long-term health benefits of their probiotic products. The incorporation of insights drawn from centenarians in the present review also highlights the unique composition of *Bifidobacterium* species in these individuals, suggesting a potential role of Bifidobacteria in longevity. Understanding the relationship between specific *Bifidobacterium* strains and the gut microbiomes of centenarians offers valuable perspectives for harnessing the therapeutic benefits of Bifidobacteria as a dietary supplement. This connection between gut Bifidobacteria and longevity underscores the importance of developing probiotic strains that benefit the general population but also contribute to healthy aging and increased lifespan, thereby reaffirming the need for ongoing research and development in this dynamic field.
